# Double Issue

**Published:** 1876

**Authors:** 


*Double Issue.*—For reasons already made known, we have thrown our January and February numbers into one issue, containing double the usual number of pages. How delighted we would be if we could, in our future monthly issues, furnish the journal in its present enlarged form; and this we could soon be able to do if each of our subscribers would regard The Record as their own, and would labor for its interests.

